# Long-Term Excellent Clinical Outcomes, High Survivorship, and Low Osteoarthritis Progression in Lateral Unicompartmental Knee Arthroplasty: A 10-Year Minimum Follow-Up

**DOI:** 10.3390/jcm14072492

**Published:** 2025-04-06

**Authors:** Matteo Marullo, Stefano Petrillo, Antonio Russo, Sergio Romagnoli

**Affiliations:** 1 Department of Joint Replacement, IRCCS Istituto Ortopedico Galeazzi, 20161 Milan, Italy; matteomarullo@hotmail.it (M.M.); sergio.romagnoli@libero.it (S.R.); 2 Humanitas Torino, Via Cellini 5, 10126 Turin, Italy; russo.antonio.92@gmail.com

**Keywords:** unicompartmental knee replacement, lateral unicompartmental knee replacement, partial knee replacement, mininvasive surgery, ACL-sparing knee replacement, knee replacement

## Abstract

**Background:** The literature on the long-term outcomes of lateral unicompartmental knee arthroplasty (UKA) remains limited due to the lower prevalence of lateral osteoarthritis (OA) and the technical challenges of the procedure. This study aimed to assess the long-term clinical outcomes, implant survivorship, and OA progression in patients undergoing lateral UKA with a minimum follow-up of 10 years. **Methods:** This retrospective study analyzed 96 lateral UKAs from 2001 to 2013 using a cemented, fixed-bearing implant. Patients with at least 10 years of follow-up were included. Clinical outcomes were measured using range of motion (ROM), a pain visual analog scale (VAS), Knee Society Scores (KSSs), and the Forgotten Joint Score (FJS). Implant survivorship was assessed using a Kaplan–Meier analysis, while OA progression in the medial compartment was evaluated radiographically. **Results:** At a mean follow-up of 14.5 years, implant survivorship was 94.7%, with five revisions primarily due to OA progression. Significant improvements were observed in ROM, VAS, and KSS (*p* < 0.01). An increase in the Kellgren–Lawrence grade in the medial compartment was reported in 47.9% of patients. **Conclusions:** Lateral UKA provides excellent long-term outcomes, demonstrating high survivorship, significant functional improvement, and high patient satisfaction.

## 1. Introduction

Valgus arthritic knees account for only about 10% of patients undergoing knee replacement, a lower proportion than varus arthritic knees [1]. This discrepancy arises because most individuals with knee osteoarthritis (OA) exhibit varus alignment in the coronal plane. Additionally, valgus alignment exerts less impact on the lateral compartment than varus alignment does on the medial compartment, making valgus arthritic knees more responsive to conservative treatments [2].

When knee replacement is necessary, the two primary options are unicompartmental knee arthroplasty (UKA) and total knee arthroplasty (TKA).

UKA is an effective treatment for isolated compartmental OA. Compared to TKA, it offers several advantages, including faster recovery, shorter hospital stays, improved range of motion (ROM), more natural knee kinematics, better functional outcomes, higher patient satisfaction, fewer complications, better cost-effectiveness, and easier revisions [3,4,5,6,7,8].

These favorable outcomes are primarily observed in medial UKA, where indications are more common and data are more widely available. In contrast, studies on lateral UKA are limited, often involving smaller patient series with shorter follow-ups.

Lateral UKA is performed far less frequently than medial UKA, accounting for only 5–10% of all UKA procedures [9,10]. Beyond the low prevalence of valgus OA, another contributing factor is the increased technical complexity of lateral UKA due to the unique anatomy and kinematics of the lateral compartment [11,12]. As a result, many surgeons prefer TKA for valgus knee as they are more familiar with the procedure.

However, with appropriate patient selection, the recent literature suggests that lateral UKA yields outcomes comparable to both medial UKA and TKA [4,8,10].

A recent systematic review by Bonanzinga et al. reported a survivorship rate of 89.8% based on seven studies with a mean follow-up of 5 to 10 years and 87.5% based on two studies with a mean follow-up of 10–15 years [9]. The authors also highlighted the low overall quality of the available literature. Similarly, Van der List et al. reached the same conclusion when comparing medial and lateral UKA survivorship, underscoring the scarcity of long-term studies on lateral UKA—particularly those extending beyond 15 years [10]. Their analysis found no significant difference in survivorship rates between medial and lateral UKA. The survivorship rates for medial UKA were 93.9% at five years, 91.7% at ten years, and 88.9% at fifteen years. For lateral UKA, the survivorship rates were 93.2%, 91.4%, and 89.4% at the same time points [10].

While extensive research is available on medial UKA, data on lateral UKA remain limited, particularly regarding long-term implant survivorship and complications. One of the most common long-term complications following lateral UKA is OA progression in the medial compartment, which is the primary reason for revision surgery and poor outcomes [2,8,9,10,13,14]. Therefore, evaluating OA progression is crucial for predicting clinical outcomes and implant longevity.

This study aimed to assess the clinical outcomes, reasons for revisions, and OA progression in the medial compartment following lateral UKA at a minimum follow-up of 10 years. It was hypothesized that high survivorship, excellent clinical outcomes, and low OA progression would be observed at long-term follow-up.

## 2. Materials and Methods

A single departmental database was retrospectively reviewed to identify all patients who underwent lateral UKA between 1 January 2001 and 31 December 2013. The study included patients who underwent isolated lateral UKA with a cemented, fixed-bearing, metal-backed prosthesis (Allegretto^®^, Zimmer Biomet Inc., Warsaw, IN, USA) and had a minimum postoperative follow-up period of 10 years. Patients were excluded if they received a different prosthesis, had incomplete medical records, or had a follow-up period of less than 10 years.

**Figure 1:**
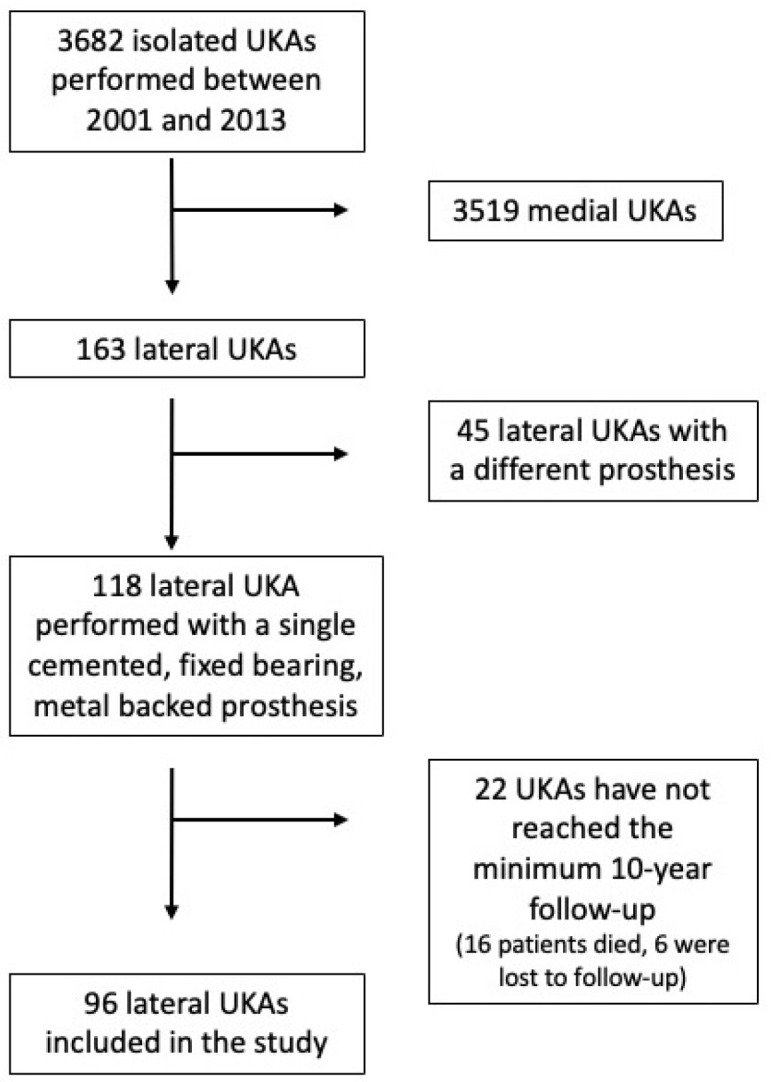
CONSORT diagram of this study.

During the study period, a total of 3682 UKAs were performed in our department, of which 163 (4.4%) were lateral UKAs. The Allegretto^®^ system was used in 118 of these cases. Twenty-two cases were excluded due to not meeting the minimum 10-year clinical follow-up requirement (sixteen patients had died, and six were lost to follow-up). Thus, 96 lateral UKAs in 88 patients were included in the study (Figure 1).

The indications for isolated lateral UKA included symptomatic lateral tibiofemoral OA (Kellgren–Lawrence [KL] grade 2 or higher [15]) and the absence of medial tibiofemoral or patellofemoral OA (KL grade lower than 2). Contraindications for lateral UKA included OA in the medial or patellofemoral compartments, inflammatory disease, clinical knee instability in the frontal or sagittal plane, a preoperative range of motion (ROM) of less than 90°, and a flexion contracture greater than 10°.

The study was approved by the ethical committee of our institution (reference number: 134/INT/2017; date of approval: 12 October 2017; clinicaltrials.gov ID: NCT04198389). All participants provided written informed consent, and the study was conducted in accordance with the STROBE Checklist for Case Series [16].

### 2.1. Surgical Technique and Rehabilitation

All patients received a cemented, fixed-bearing, metal-backed, resurfacing lateral UKA (Allegretto^®^, Zimmer Biomet Inc., Warsaw, IN, USA) through a minimally invasive lateral parapatellar approach. Tourniquet was never used. A moderate under correction of the deformity was the aim of the alignment after UKA [14]. The senior author performed all surgeries.

On the first postoperative day, all patients began progressive weight-bearing. Passive and active range-of-motion (ROM) exercises were initiated within 12 h after the surgery. Patients were discharged on the second postoperative day once they could walk independently using crutches and achieved a minimum of 90° knee flexion. Full weight-bearing was permitted after the first week, although patients were advised to use crutches for the first three weeks post-surgery. Everyday activities were resumed 30 days after surgery, although muscle strengthening continued for 60 to 75 days postoperatively.

### 2.2. Clinical and Radiographic Evaluations

Preoperative patient characteristics included gender, age at the time of surgery, body mass index (BMI), the degree of tibiofemoral and patellofemoral OA based on the Kellgren and Lawrence classification [15], and preoperative knee range of motion and pain intensity measured using a visual analog scale (VAS). Postoperative follow-ups were conducted at 3 months, 12 months, and annually thereafter. Each follow-up assessment included radiological and clinical evaluations, along with a questionnaire on patient satisfaction.

Radiographic evaluation, performed both preoperatively and postoperatively, consisted of a full-length standing radiograph of the lower limb, a standing posterior–anterior radiograph of both knees at 45° knee flexion (Rosenberg view [17]), a true lateral view, and a 30° patellar axial view.

The mechanical axis of the lower limb was assessed using a long-standing radiograph by measuring the angle between two reference lines: one connecting the center of the femoral head to the center of the knee and another extending from the center of the knee to the center of the talus (hip–knee–ankle angle, HKA). A mechanical axis exceeding 180° was categorized as valgus alignment. All radiographic data were digitally analyzed and measured using the Picture Archiving and Communication System (Philips Medical Systems; Sectra-Imtec AB, Linköping, Sweden). Two independent observers, who were not involved in the surgical procedures, performed the evaluations.

Clinical outcome scores were assessed preoperatively and at each follow-up visit. These included the Knee Society Score (KSS) [18], both clinical and functional components (KSS-C and KSS-F, respectively), the Tegner Activity Scale (TAS) [19], and the University of California Los Angeles (UCLA) Activity Score [20]. The Forgotten Joint Score (FJS) was introduced in 2020 following the validation of its national version [21].

Patients rated their overall satisfaction with the procedure on a scale ranging from 1 (very satisfied) to 5 (very dissatisfied). Those who underwent bilateral procedures provided independent satisfaction ratings for each side. Patient-reported outcome measures (PROMs) at the final follow-up were compared between patients with obesity (BMI > 30 kg/m^2^) and non-obese patients (BMI < 30 kg/m^2^).

Implant failure was defined as any subsequent surgical intervention following the initial procedure, including implant removal, revision to total knee arthroplasty (TKA) for any reason, or the placement of a UKA or patellofemoral prosthesis in the same knee due to OA progression.

### 2.3. Statistical Analysis

Data analysis was conducted using IBM SPSS version 26.0 (IBM Corp., Armonk, NY, USA). Continuous variables were reported as mean, range, and standard deviation (SD). Categorical variables were expressed as the number of cases and percentage. Distribution of data was assessed using the Shapiro–Wilk text. Baseline values of ROM and patient-reported outcome measures (PROMs) were compared to the values obtained at the latest follow-up through the paired *t*-test. Kaplan–Meier curves were used to assess failure-free survival. Failure was defined as the revision of the lateral UKA or any re-operation due to OA progression. The preoperative level of OA in the medial compartment was compared to that recorded at the final follow-up using the Wilcoxon signed rank test. A binomial logistic regression analysis was conducted to ascertain the possible influences of age, gender, BMI, and final HKA on OA progression. Statistical significance was set at *p* < 0.05 for all variables studied.

## 3. Results

A total of 96 lateral UKAs in 88 patients were included in the study. The mean age was 64.6 ± 10.9 years (range, 42 to 89). The mean follow-up was 14.5 ± 3.3 (range of 10 to 22.4) years. Sixty-five patients (73.9%) were women. Patient demographics are shown in Table 1.

### 3.1. Survival Analysis

**Figure 2:**
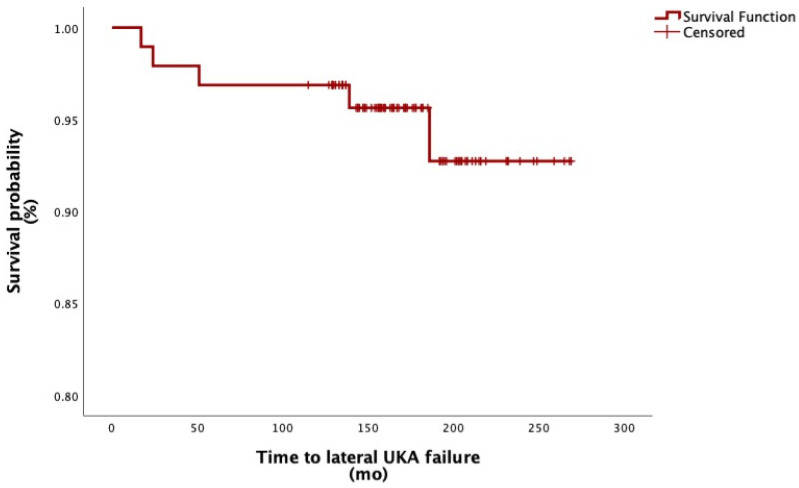
Survival function of lateral UKA; mo, months.

At the latest follow-up, five lateral UKAs underwent implant failure, which corresponds to a survival rate of 94.7% (Figure 2). Of these, two (2.1%) were converted to TKA for OA progression, one (1.0%) was revised with TKA for polyethylene wear, one (1.0%) was revised with TKA for loosening of the tibial component, and in one case (1.0%), OA progression in the medial compartment was managed with the addition of a medial UKA.

The Kaplan–Meier analysis estimated the 5-, 10-, and 15-year survival probability of implants to be 97.3%, 95.9%, and 93.1%, respectively. The mean time from index surgery to failure was 9.0 ± 6.3 (range of 3 to 18) years.

### 3.2. Progression of OA in Medial Compartment

A total of 46 (47.9%) patients presented at the latest follow-up with a level of OA in the medial compartment higher than that registered preoperatively. Of these, sixteen (16.7%) increased from K-L 0 to 1; one (1.0%) increased from K-L 0 to 3; one (1.0%) increased from K-L 0 to 4; twenty-one (21.9%) increased from K-L 1 to 2; two (2.1%) increased from K-L 1 to 3; one (1.0%) increased from K-L 1 to 4; and four (4.2%) increased from K-L 2 to 3.

The median increase in K-L classification was 1 unit.

**Figure 3:**
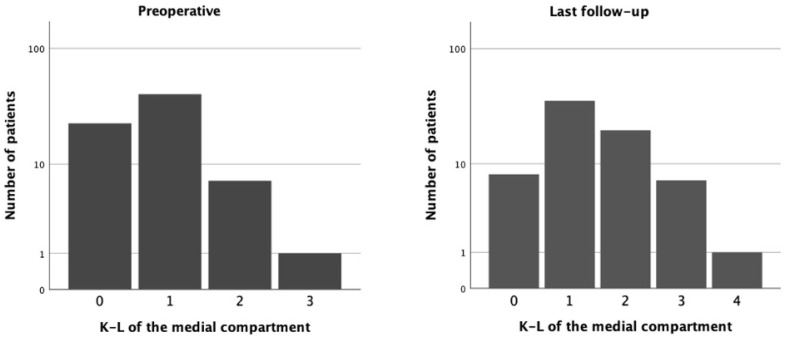
Kellgren–Lawrence grade in medial compartment. (**Left**) Preoperative grade. (**Right**) Grade at last follow-up (mean 14.5 years).

The difference in the preoperative and last follow-up levels of medial OA, as expressed by K-L, was considered significant (*p* < 0.01). See Figure 3.

Nonetheless, OA progression in the medial compartment did not lead to any clinical impairment, except in the three patients who required revision surgery due to disease progression. However, none of the patients who experienced OA progression reported dissatisfaction with their knees.

No correlation was found between medial OA progression and age (*p* = 0.29), gender (*p* = 0.53), BMI (*p* = 0.42), and final HKA (*p* = 0.09).

### 3.3. Functional Outcomes

The following variables significantly improved from baseline to values registered at the last follow-up: ROM (*p* < 0.01), VAS (*p* < 0.01), KSS-K (*p* < 0.01), and KSS-F (*p* < 0.01). No significant difference was found between the preoperative and last follow-up values of the TAS (*p* = 0.46) and UCLA (*p* = 0.52). Details are shown in Table 2.

A comparison of the functional outcomes between patients with obesity and non-obese patients showed a significant difference only in the preoperative values of ROM, which did not differ significantly at final follow-up (Table 3).

### 3.4. Satisfaction

**Figure 4:**
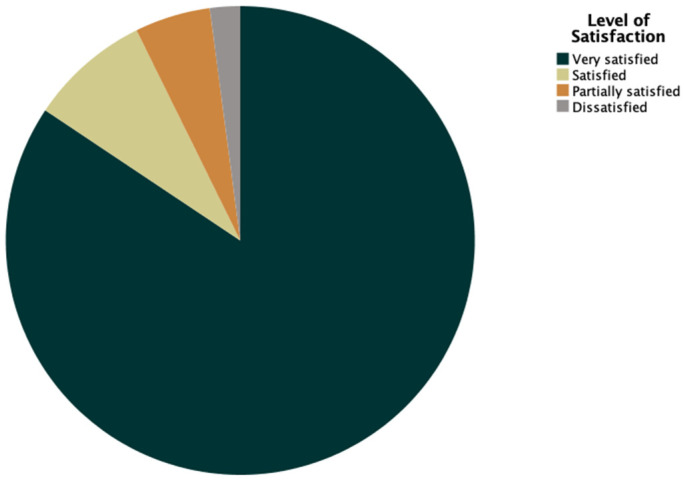
Pie chart representing patients’ satisfaction.

At the latest follow-up, 81 patients (84.4%) were very satisfied with the procedure. Eight patients (8.3%) were satisfied (level of satisfaction 2), five were (5.2%) partially satisfied (level 3), and two (2.1%) were dissatisfied (level 4). See Figure 4.

No difference in the level of satisfaction was found between patients with obesity and non-obese patients (*p* = 0.19).

Among patients who eventually underwent revision of their lateral UKA, two (2.1%) were partially satisfied (level 3) with the procedure, two (2.1%) were satisfied, and the remaining two (2.1%) patients stated that they were overall very satisfied. One patient who was dissatisfied had severe progression of patellofemoral OA but refused revision.

### 3.5. Complications

A total of four (4.2%) patients experienced complications. Two cases (2.1%) of intra-operative periprosthetic fractures were registered. One involved the lateral femoral condyle, and it was fixated with two screws; the other one was a tibial plateau fracture, and osteosynthesis was achieved using three screws. None of these two patients experienced implant failure or revision at a mean final follow-up of 13.2 years.

One patient (1.0%) presented end-stage OA in the medial compartment 13.3 years after index surgery, but he was satisfied with his knee and refused revision. One patient (1.0%) presented with patellofemoral OA 11.1 years after lateral UKA; revision surgery was proposed but she refused, even though she was dissatisfied with the outcome of the procedure.

## 4. Discussion

The main finding of the present study was that lateral UKA is an effective treatment for end-stage lateral OA, with excellent clinical and functional results lasting even at long-term follow-up with low evidence of OA progression in the medial compartment and thus high survivorship at a minimum follow-up of 10 years (94.7% survivorship at a mean of 14.5 years).

Data on the long-term outcomes following lateral UKA are limited. This research gap may be due to two primary factors: the less frequent surgical indication for lateral UKA, as isolated lateral OA is less common than isolated medial OA or diffuse OA, and the technical challenges of lateral UKA, which lead many surgeons to prefer TKA [1,2,9,10,11,12].

TKA has demonstrated excellent long-term outcomes in the treatment of valgus knee OA. Tucker et al. evaluated 275 TKAs in patients with severe valgus deformities (≥10°) over a mean follow-up period of 10.4 years [22]. A total of 78% of patients reported good to excellent results, with a mean Oxford Knee Score of 27.8 and an American Knee Society clinical score of 85.6. The revision rate was 0.36%, with a reoperation rate of 4.73%. The 10-year Kaplan–Meier implant survival rate was 99.6%.

The main weakness of lateral UKAs was supposed to be their limited survivorship compared to TKAs [4,6,8]. However, modern lateral UKAs have demonstrated average survival rates ranging from 93 to 96% at 5 years and ranging from 89 to 93% at 10 years [11,12,14,23].

A recent systematic review examined 47 articles about lateral UKA [9], demonstrating survivorship and satisfaction rates of lateral UKA comparable to those of medial UKA and TKA. Nonetheless, the mean follow-up of the studies examined was only 60 months, and the literature on the long-term follow-up of lateral UKA is still very limited.

Our study stands out because it analyzes the clinical and functional outcomes of a fixed-bearing, metal-backed tibial component lateral UKA performed using a conventional technique, and it assesses the long-term progression of osteoarthritis in the medial compartment.

In our series, all clinical and functional outcomes, except for the TAS and UCLA score, showed significant improvement from baseline to the final follow-up. The lack of improvement in these two functional scores may be attributed to the aging population over the extended follow-up period.

Plancher et al. presented the clinical results of 61 cemented, fixed-bearing, metal-backed tibial component lateral UKAs performed without robotic assistance at a mean follow-up of 10 years (range, 4–17) [24]. Three patients (4.9%) had revision to TKA, leading to a 10-year survivorship of 95.1%.

Ruderman et al. recently published a study reporting the 10-year follow-up results of 77 cemented, fixed-bearing, metal-backed lateral UKAs implanted using robotic arm assistance [25]. They reported five revisions (two due to OA progression, two for unexplained pain, and one for infection), resulting in a 10-year survival rate of 96.1%. Unfortunately, their study did not include clinical or functional outcomes. Nevertheless, their survival rate is comparable to that of our series, which was performed using a conventional technique.

A recent retrospective study analyzed the outcomes of 84 robotic-assisted lateral UKAs with a mean follow-up of 4.0 years (range 2.0–7.0) [26]. The five-year implant survivorship was 92.9% for all-cause reoperation and 100% for conversion to TKA, decreasing to 88.9% at six years. Patients reported high satisfaction (4.7/5) and an average Forgotten Joint Score-12 of 82.7/100.

Robotic assistance in UKA has been shown to enhance surgical precision and improve implant positioning compared to manual techniques [27]. However, it has not yet been definitively associated with better clinical outcomes over conventional methods [27]. Future long-term studies should evaluate functional outcomes using measures with minimal ceiling effects to determine whether the increased accuracy of robotic technologies leads to superior clinical results.

In 2019, Deroche et al. presented the results of 39 fixed, all-polyethylene tibial bearing lateral UKAs at a mean follow-up of 17.9 years [28]. A total of 8 out of 39 knees (20.5%) experienced revision. The main reason for revision was OA progression (87.5%), followed by aseptic loosening of the tibial component (12.5%). The mean functional and clinical Knee Society Scores at the last follow-up were 66.5 and 84.4, respectively.

In 2024, the same group published the results of 28 lateral UKAs at a mean follow-up of 22 years [29]. The implant used was the same, namely a cemented, fixed-bearing, all-polyethylene tibial component. Eight knees (28.6%) underwent revision surgery: five for OA progression, two for aseptic loosening of the tibial component, and one for poly wear. At the last follow-up, the mean functional Knee Society Score was 41.5, and the mean objective KSS score was 79.4.

Murray et al. published the long-term results of a case series of 82 lateral UKAs; even in this case, the implants were cemented, fixed-bearing, all-polyethylene tibial component UKAs [30]. They demonstrated a survivorship of 72% at 15 years of follow-up. The most common cause of revision in their series was also OA progression.

A recent meta-analysis compared the failure rates of different prosthetic designs in lateral UKAs [23], demonstrating that fixed-bearing, metal-backed UKAs have a significantly lower failure rate compared to fixed-bearing, all-polyethylene or mobile-bearing, metal-backed ones (0.8% vs. 8.6% and 7.1%, respectively). No significative difference among groups was detected when comparing all implants with regard to the follow-up time.

In our series, the primary failure mode of lateral UKA was OA progression in the medial compartment, responsible for three out of five failures. The remaining two failures were due to tibial component loosening, which occurred three years postoperatively, likely due to an improper cementation technique, and polyethylene wear, which developed 18 years after surgery—an expected outcome given the long postoperative duration.

Like in our series, the predominant failure mode of lateral UKA observed was OA progression requiring medial UKA or TKA [28,29,30,31]. It differs from medial UKA, in which the main cause of failure is aseptic loosening [32]. A possible explanation is that patients who had varus alignment have the load transferred through the medial compartment both in the static and dynamic phase. Instead, patients who had valgus alignment have the load in the lateral compartment only during the static phase; during the dynamic phase, the load shifts through the medial compartment. The higher load in the medial compartment during the dynamic phase of gait in both varus and valgus alignment patients may favor aseptic loosening in medial UKA and OA progression in lateral UKA [14]. A clear postoperative valgus alignment (HKA ≥ 184°) appeared to positively influence the survival rate of lateral UKA more than a mild valgus alignment (HKA < 184°) [14].

While our study provides robust long-term data, it is essential to acknowledge its limitations. The retrospective design and relatively small sample size, inevitable given the low prevalence of isolated lateral OA, may affect generalizability. Additionally, the lack of a direct comparison group (e.g., TKA or robotic-assisted UKA) limits the scope for comparative analysis. Future research should prioritize randomized controlled trials and larger multicenter cohorts to further validate these findings.

## 5. Conclusions

In conclusion, lateral UKA with a cemented, fixed-bearing, metal-backed implant provides excellent long-term survivorship and functional outcomes with minimal complications. These findings contribute valuable evidence to the limited literature on lateral UKA, supporting its use as a durable solution for lateral compartment OA when appropriate indications are met.

## Figures and Tables

**Table 1:** Demographic characteristics of patients. BMI, body mass index; SD, standard deviation.

Variable		Number of Cases (%)	Mean ± SD
Gender	men	23 (26.1)	/
	women	65 (73.9)	/
Age, years		96 (100)	64.6 ± 10.9
BMI, kg/m^2^		96 (100)	26.8 ± 4.2
Follow-up, years		96 (100)	14.5 ± 3.3

**Table 2:** Clinical functional outcomes.

Variable	Preoperative	Last FU	*p*-Value
ROM, °	109.1 ± 9.4	126.6 ± 6.9	<0.01
VAS, points	5.0 ± 1.4	1.7 ± 1.1	<0.01
TAS, points	2.7 ± 0.8	2.7 ± 0.8	0.44
UCLA, points	4.8 ± 1.5	4.8 ± 1.6	0.41
KSS-K, points	58.3 ± 9.4	87.7 ± 10.9	<0.01
KSS-F, points	58.8 ± 14.9	82.6 ± 15.6	<0.01
FJS, points	/	97.4 ± 8.6	/

FU, follow-up; KSS-K, Knee Society Score-Clinical; KSS-F, Knee Society Score-functional; FJS, Forgotten Joint Score; ROM, range of motion; TAS, Tegner Activity Scale; UCLA, University of California Los Angeles activity scale; VAS, visual analog scale for pain. Note: *p*-value is for paired *t*-test.

**Table 3:** Comparison of outcomes between patients with obesity and non-obese patients.

Variable	Non-Obese (*n* = 74)	Patients with Obesity (*n* = 22)	*p*-Value
Preoperative ROM, °	110.1 ± 9.5	105.6 ± 8.1	0.04
Last follow-up ROM, °	126.5 ± 7.5	126.8 ± 4.5	0.84
Preoperative VAS, points	4.9 ± 1.4	5.2 ± 1.1	0.28
Last follow-up VAS, points	1.5 ± 0.9	1.9 ± 1.2	0.13
Preoperative TAS, points	2.7 ± 0.8	2.4 ± 0.9	0.11
Last follow-up TAS, points	2.7 ± 0.9	2.6 ± 0.8	0.96
Preoperative UCLA, points	4.9 ± 1.6	4.6 ± 1.1	0.43
Last follow-up UCLA, points	4.9 ± 1.7	4.7 ± 1.1	0.81
Preoperative KSS-K, points	57.5 ± 9.9	61.3 ± 6.3	0.10
Last follow-up KSS-K, points	87.8 ± 10.9	87.3 ± 11.5	0.85
Preoperative KSS-F, points	57.4 ± 16.1	63.6 ± 8.9	0.09
Last follow-up KSS-F, points	82.4 ± 16.3	83.1 ± 13.6	0.87
Last follow-up FJS, points	96.3 ± 9.7	99.3 ± 2.3	0.16

KSS-K, Knee Society Score-Clinical; KSS-F, Knee Society Score-functional; FJS, Forgotten Joint Score; ROM, range of motion; TAS, Tegner Activity Scale; UCLA, University of California Los Angeles activity scale; VAS, visual analog scale for pain. Note: *p*-value is for *t*-test for independent means.

## Data Availability

Data supporting the reported results can be found in database generated during the study.
